# Mechanisms of immune evasion and current status of checkpoint inhibitors in non‐small cell lung cancer

**DOI:** 10.1002/cam4.819

**Published:** 2016-07-15

**Authors:** Angel Qin, David G. Coffey, Edus H. Warren, Nithya Ramnath

**Affiliations:** ^1^ Division of Hematology and OncologyDepartment of MedicineUniversity of MichiganAnn ArborMichigan; ^2^ Clinical Research DivisionFred Hutchinson Cancer Research CenterSeattleWashington; ^3^ Division of Medical OncologyDepartment of MedicineUniversity of WashingtonSeattleWashington; ^4^ VA Ann Arbor Health Care SystemAnn ArborMichigan

**Keywords:** Determinants of response, immune evasion, non‐small cell lung cancer, PD‐1, PD‐L1

## Abstract

In the past several years, immunotherapy has emerged as a viable treatment option for patients with advanced non‐small cell lung cancer (NSCLC) without actionable driver mutations that have progressed on standard chemotherapy. We are also beginning to understand the methods of immune evasion employed by NSCLC which likely contribute to the 20% response rate to immunotherapy. It is also yet unclear what tumor or patient factors predict response to immunotherapy. The objectives of this review are (1) review the immunogenicity of NSCLC (2) describe the mechanisms of immune evasion (3) summarize efforts to target the anti‐program death‐1 (PD‐1) and anti‐program death‐ligand 1(PD‐L1) pathway (4) outline determinants of response to PD‐1/PD‐L1 therapy and (5) discuss potential future areas for research.

## Introduction

Lung cancer remains the leading cause of cancer‐related death in the United States. The majority of patients present with advanced non‐small cell lung cancer (NSCLC), for which the 5‐year survival is less than 15% 1. In recent years, high‐throughput DNA sequencing has demonstrated that NSCLC is not a single, homogeneous disease entity, but rather a collection of neoplasms with distinct profiles of molecular abnormalities. This has shifted the landscape of NSCLC therapy to a personalized approach that is driven by molecular alterations present in each patient's tumor, leading to improved survival for some patients. Nonetheless, an “actionable” genetic driver alteration cannot be identified for a significant fraction of patients with metastatic NSCLC, and these patients have a median overall survival of only 1 year 2.

Beyond surgery, radiation, and chemotherapy, immunotherapy has emerged in recent years as a fourth pillar in the therapeutic approach against lung cancer. By harnessing the native antitumor immune response, this approach is more physiologic and agnostic to the histological type of lung cancer. Immune checkpoint inhibition aims to counteract mechanisms of immune tolerance co‐opted by many cancers. Immune checkpoint inhibitors provide a physiological way of unleashing the adaptive immune response. However, there is a critical need to identify patients who will most likely respond to this approach and convert more patients to durable responders leading to improved survival.

### Immunogenicity of NSCLC

For the immune system to recognize tumor cells, it must differentiate them from the normal cells from which they arise. A cancer cell may produce an antigen recognizable by the immune system by overexpression of self‐proteins not usually expressed in most of the body or through formation of a neoantigen. A neoantigen is derived from a nonsynonymous mutation or through aberrant transcription, incomplete splicing, translation of alternative or cryptic open‐reading frames, or posttranslational modifications. Neoantigen formation is a probabilistic event which depends on the mutational load involving the number of nonsynonymous mutations. If a cell acquires an immunogenic mutation, then it may be sought out and destroyed by the host immune system in a process known as immunosurveillance 3. Owing to the mutagenicity of tobacco smoke, it is of no surprise that NSCLCs are among the tumors with the highest frequency of somatic mutations only surpassed by melanoma 4. Until recently, NSCLC was felt to be poorly immunogenic as it was thought that NSCLC‐related antigens did not efficiently prime an antitumor immune response.

Attempts to prime the immune response in NSCLC included using interleukin‐2 (IL‐2) and tumor necrosis factor (TNF). These cytokines boost natural killer (NK) cell activity and activate macrophages, two processes essential to the activation of an innate immune response. Motivated by favorable responses in patients with melanoma and renal cell carcinoma, combination IL‐2 and TNF therapy was used in patients with advanced NSCLC. However, these treatments failed to produce disease response and were associated with significant toxicities 5. Similarly, addition of interferon‐*α* therapy to chemotherapy failed to demonstrate improved disease response, again associated with significant toxicities 6.

Therapeutic vaccinations to prime the immune system against tumor‐specific antigens have also been attempted. These strategies have targeted neoantigens or self‐proteins that are overexpressed or tissue‐specific gene products. For example, belagenpumatucel‐L is a vaccine derived from four irradiated NSCLC tumor cell lines that was tested in a phase II trial and demonstrated safety and efficacy in low volume disease 7. However, a phase III trial in patients with advanced disease did not reveal improved overall survival (OS) when using it as a maintenance therapy compared to placebo 8. A phase III trial involving a vaccine against MAGE‐A3 (expressed in 35–50% of NSCLC cells) also failed to reveal significant improvements in disease‐free survival (DFS) or OS 9. The results of these studies suggest that vaccines directed against common NSCLC epitopes may not be effective alone for the treatment of the disease since we now know that tumor has also evolved mechanisms to evade the immune response.

### Mechanisms of immune evasion and promotion of tolerance by NSCLC

T lymphocytes in conjunction with antigen‐presenting cells (APCs) such as macrophages and dendritic cells are responsible for antigen‐specific cell‐mediated immunity. Tumor‐derived antigen peptides are displayed on the surface of the APCs via the major histocompatibility complex class II (MHCII). The activation of CD4+ T helper cells by the APCs help to bolster and maintain the CD8+ cytotoxic T lymphocyte (CTL) response through the production of cytokines such as IL‐2. CTLs can also interact directly with tumor cells via their major histocompatibility complex class I (MHCI). Regardless of the mechanism of activation, CTLs initiate target cell killing via the release of cytotoxic granules or inducing target cell apoptosis. The importance of CTLs in suppressing tumor growth is demonstrated by animal studies mimicking aggressive human lung cancers in which mice deficient in CD8+ T cells had increased tumor burden, quicker acceleration to end‐stage disease, and decreased survival 10.

For there to be a successful T‐cell response that ultimately leads to cancer regression, three steps must occur: (1) APCs must present tumor antigen and activate an effector T‐cell response (2) primed T cells must successfully home in on and infiltrate stromal tissue prior to binding to their target on the tumor, and (3) the T‐cell receptors (TCRs) of the infiltrating T cells must bind to the MHCI–peptide complex to activate the cytotoxic T‐cell response 11. Lung cancer cells have developed mechanisms to evade immune detection and activation through blocking crucial steps in the generation of this cytotoxic T‐cell response.

#### Antigen presentation

Though the mechanism of downregulation is unclear, Foukas et al. showed that there was significantly reduced MHCII expression by APCs in 78% of NSCLC tumor samples they examined 12. They hypothesized that this decrease may be due to the inhibitory effects of TGF*β* and IL‐10 secreted by NSCLC tumor cells. Lung cancer cells themselves also present endogenous antigens via MHCI. Studies show that NSCLC tumor cells can also escape this key step of immune recognition by downregulating or altering their MHCI expression 13, 14. The expression of other components of the antigen presentation pathway such as *β*‐microglobulin and transporter associated with antigen processing (TAP1 and TAP2) are also significantly decreased in NSCLC 15. Especially pertinent to NSCLC, tobacco exposure has been shown to decrease expression of MHCI and TAP1 protein 16.

#### Tumor‐infiltrating T‐cell phenotypes

The immune evasive measures utilized by NSCLC tumor cells can be broadly separated into two categories as defined by cellular and molecular characteristics in the tumor microenvironment—an inflamed T‐cell phenotype that actively suppresses immune activation and a noninflamed phenotype that passively escapes immune detection 17. The inflamed phenotype is characterized by tumor infiltration by CD8+ T cells. It is not well known what signals attract activated T cells to the tumor as tumor‐infiltrating lymphocytes (TILs) do not accumulate in all tumors. There is evidence that T‐cell homing is likely driven by the expression of certain chemokines, which are secreted by the stromal elements and tumors themselves 18. Once tumor infiltration occurs, the cytokine milieu and the cellular composition of the tumor microenvironment interact to facilitate or inhibit tumor growth. In the inflamed phenotype, CD4+ helper T cells augment the immune response by releasing cytokines such as IFN*γ* and TNF, which boost the cytotoxic CD8+ T‐cell response 19. Concomitant infiltration by both CD4+ T cells and CD8+ T cells have been shown to portend favorable prognosis in NSCLC patients 20.

**Figure 1:**
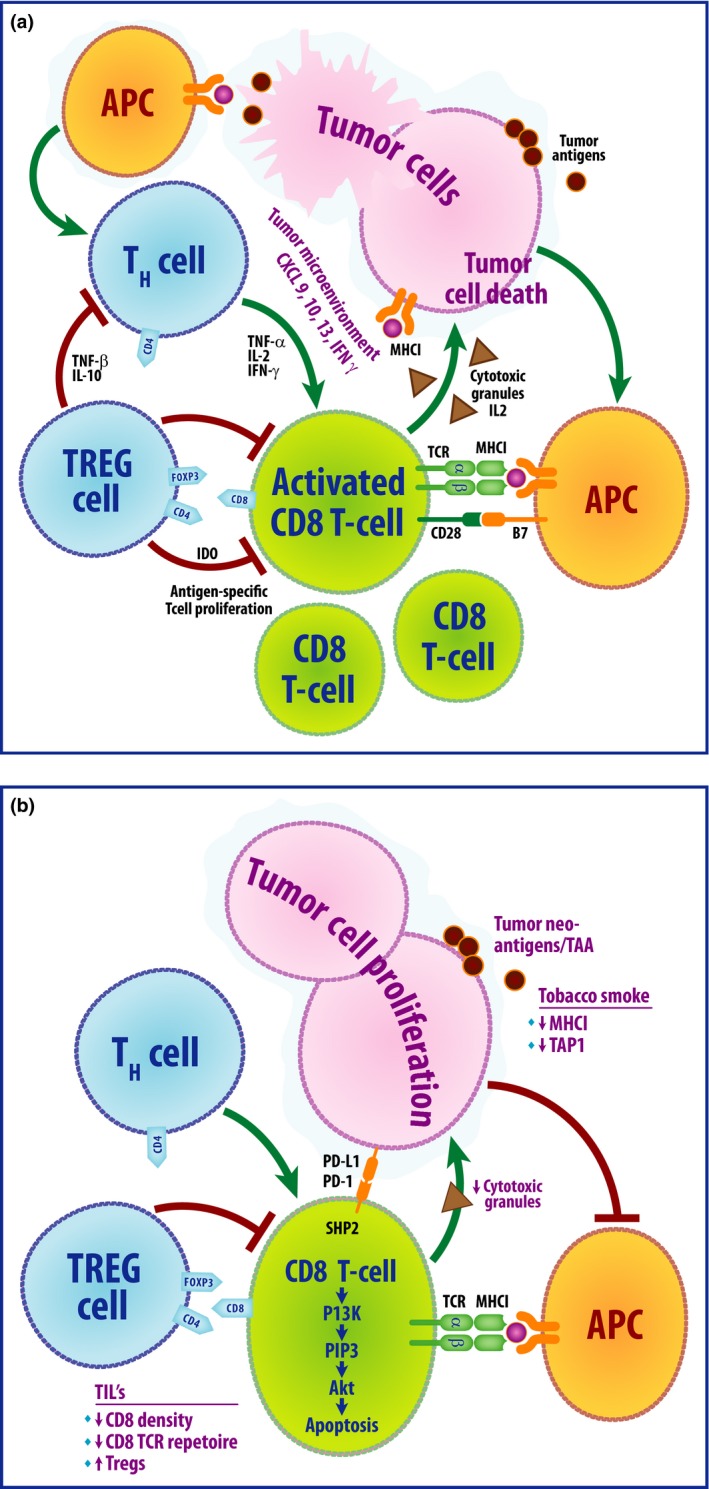
Top panel (A) depicts the active antitumor immune response and the interactions within the tumor microenvironment that lead to tumor cell apoptosis. Tumor antigen presentation by APCs stimulates activation of CD8+ CTLs through interaction of MHCI/TCR and B7/CD28; in turn, this leads to activation of pathways involved in CTL proliferation and IL‐2 production. The APCs also stimulate proliferation of T helper (Th) cells that secrete activating cytokines such as TNF *α* and IL‐2 which further promote CTL activation and proliferation. CTLs ultimately secrete cytotoxic granules that result in tumor cell death. As a countermeasure, the tumor cells secrete cytokines such as TGF *β* and IL‐10 that stimulate FOXP3+ Treg proliferation. Tregs play a crucial role in dampening the immune response through inhibition of CTLs and Th cells. Bottom panel (B) depicts breakdown of the immune response through various evasive mechanisms employed by tumor cells that lead to tumor progression. Alterations of the MHCI and downregulation of TAP1 by tobacco smoke hinder APC function. FOXP3+ Tregs bind to activating cytokines such as IL‐2, thereby limiting CTL proliferation and activation. An increased density of FOXP3+ Treg cells and decreased CTLs is thought to contribute to tumor cell proliferation. APC, antigen presenting cells; CTL, cytotoxic T lymphocyte; TNF, tumor necrosis factor.

As a countermeasure, NSCLC tumor cells secrete cytokines such as IL‐10, which promotes regulatory T‐cell (Treg) proliferation and suppresses CD8+ T‐cell‐mediated cytotoxic killing 19. NSCLC tumors also have elevated expression of the chemokine CCL20, which aids in the recruitment of FOXP3+ Treg cells into the tumor microenvironment 21. Tregs play a crucial role in immune homeostasis by allowing tolerance and preventing autoimmunity through suppression of CD8+ T cells. Tregs induce a dysfunctional state in tumor‐infiltrating CTLs that resembles T‐cell exhaustion, characterized by low expression of effector cytokines and inefficient cytotoxic granule release. FOXP3 is a member of the forkhead or winged helix family of transcription factor and is a surface marker of suppressive Treg cells. In NSCLC, tumor cells secrete the cytokine TGF*β*, which promotes maturation of Treg cells into the CD25+FOXP3+ phenotype 22, contributing to immune quiescence. Studies have shown that patients with NSCLC have higher numbers of CD25+FOXP3+ Treg cells in the tumor itself 23 as well as in the peripheral blood 24. Depletion of FOXP3+ Treg cells in a NSCLC tumor model resulted in significant decrease of tumor burden 10. In NSCLC, increased tumor infiltration with CD8+ T cells is associated with improved survival 25, whereas higher infiltration by FOXP3+ Treg cells is associated with disease recurrence 26. This balance between the immune‐activating and the immune‐suppressing forces in the tumor microenvironment results in tumor regression or progression (Fig. 1).

The noninflamed phenotype is characterized by poor tumor infiltration by T lymphocytes and immune tolerance 17. Due to unknown mechanisms, some tumors do not attract T cells and largely grow unhindered by the immune system. Tregs can express a heterotrimeric receptor that binds IL‐2 with a 100‐fold higher affinity compared to the dimeric receptor, thereby effectively acting as “competitive sinks” for IL‐2 and thereby inhibiting the cytotoxic T‐cell inflammatory response 27. Tregs can also act as sinks for crucial homing cytokines such as IL‐7 and IL‐5, thereby preventing T‐cell tumor infiltration 28. In one series, about 35% of NSCLC tumors demonstrated absent or only moderate CD8+ T‐cell infiltration 29. Recent studies also show that other cellular components of the tumor microenvironment such as macrophages and NK cells play a key role in suppressing inflammation 30. Of special interest are tumor‐associated macrophages (TAMs), in particular the M2 phenotype, which are present in the tumor microenvironment stroma, and have been shown to promote tumor growth by enhancing tumor cell proliferation and metastasis, as well as suppressing the antitumor immune response in NSCLC cancer 31.

#### Alterations of the TCR complex

In a mouse lung cancer model, tumor‐associated myeloid cells were shown to produce elevated levels of the enzyme arginase I, which decreases expression of the CD3*ζ* chain within the TCR complex. In the same study, Rodriguez et al. showed that human NSCLC tumors also contained elevated expression of arginase I 32. As in the mouse model, the TILs in the human samples also had markedly decreased expression of the CD3*ζ* chain. Nagaraj et al. showed in *in vivo* mouse models that tumor‐associated myeloid cells can modify the TCR via generation of reactive oxygen species and peroxynitrite resulting in loss of ability of CD8+ T cells to bind to MHC, thereby inhibiting the antigen‐specific T‐cell response 33.

### Immune checkpoint inhibition

In the process of activation of CD8+ T cells, additional costimulatory signals are exchanged in conjunction with the binding of the TCR to the MHC–peptide complex on the APC. These signals can be either activating or inhibitory in nature. A balance between these signals is crucial to the functioning of the immune system, allowing defense against a variety of pathogens while simultaneously allowing tolerance of self. The most well‐characterized stimulatory interaction is between the B7 family of molecules (B7.1 and B7.2 also known as CD80 and CD86 respectively) expressed on APCs and activated B cells and CD28 expressed on T cells 34. CD80 and CD86 also interact with CTLA‐4 on T cells, whose expression is induced by T‐cell activation. CTLA‐4 is one of the key immune checkpoint inhibitor molecules, which allow self‐tolerance and prevent autoimmunity by regulating and limiting T‐cell activation. In knockout mice studies, absence of CTLA‐4 signaling lead to constitutive activation of ZAP‐70 35. CTLA‐4 recruits two phosphatases, SHP2 and PP2A, to dampen the signals propagated by the TCR.

The program death‐1 (PD‐1)/program death‐ligand 1(PD‐L1) pathway is another crucial self‐tolerance pathway that tumor cells have hijacked to escape immune elimination. PD‐1 (CD279) is a glycoprotein that has an Ig variable‐type like domain at the extracellular terminus and an immunoreceptor tyrosine‐based switch motif at the cytoplasmic terminus 36. PD‐1 is expressed by activated B cells, T cells, NK cells, monocytes, and some dendritic cells and its major ligands are PD‐L1 and PD‐L2. While PD‐L2 binds to PD‐1 with three times more affinity compared to PD‐L1, it is expressed by fewer cell types, including normal pulmonary alveolar epithelial cells. In contrast, PD‐L1 is constitutively expressed by B cells, dendritic cells, macrophages as well as nonhematopoietic cells such as vascular endothelial cells, neurons, and certain epithelial cells. In the T cell, the binding of PD‐L1 to PD‐1 inhibits phosphatidylinositol‐3‐kinase (PI3K) and therefore, the Akt pathway, which is essential for cell proliferation and survival 37. This leads to a decrease in the number of T cells. Binding of PD‐1 to PD‐L1 also blocks the synthesis and secretion of IL‐2, which is crucial for effector T‐cell differentiation 38. PD‐1 signaling can also lead to the dephosphorylation of the CD3*ζ* chain, thereby dampening TCR signaling 39.

Tumoral expression of PD‐L1 takes advantage of PD‐1 expression by T cells to promote immune tolerance. Compared to healthy tissue, PD‐L1 expression has been shown to be increased in NSCLC tumor samples when assessed by immunohistochemical staining 40, 41. Tumor‐infiltrating CD8+ T cells in NSCLC express higher levels of PD‐1 compared to CD8+ T cells in peripheral blood from the same patient 42. In the same study, Zhang et al. showed that functionally, these PD‐1+ CD8+TILs produced less IFN*γ* and IL‐2 and proliferated less in response to stimulation, revealing a state of dysfunction 42. Examining tissue and blood samples from patients with different tumors treated with anti‐PD‐1 therapy including NSCLC, PD‐1 expression by TILs was significantly positively correlated with PD‐L1 expression by the tumor cells and immune infiltrate cells, reflecting an immunosuppressive microenvironment 43. In the same study by Taube et al.*,* higher tumor expression of PD‐L1 was also a positive correlate for response to anti‐PD‐1 therapy.

#### Immune checkpoint inhibitors and NSCLC

With the advent of checkpoint inhibitors, including PD‐1 and PD‐L1 inhibitors, there has been a paradigm shift in immunotherapy from targeting the cancer cell to targeting tumor‐mediated immune tolerance 44. As such, there has been a resurgence of interest in applying immunotherapy to treat NSCLC.

Ipilimumab, a monoclonal antibody against CTLA‐4, was the first checkpoint inhibitor to be FDA approved after a phase III trial demonstrated improved median overall survival in patients with stage III/IV previously treated unresectable melanoma when used in conjunction with a glycoprotein 100 vaccine compared to vaccine alone 45. Lynch et al. assessed the activity of ipilimumab given in conjunction with carboplatin and paclitaxel in a concurrent or phased regimen in patients with previous untreated stage IIIB/IV NSCLC in a phase II randomized trial 46. The phased regimen group had improved median PFS compared to chemotherapy alone at 5.7 months compared to 4.6 months, *P* = 0.05. There was no improvement in the concurrent regimen group. The authors did note that the improved efficacy with the addition of ipilimumab was for patients with squamous NSCLC. A recently published phase I trial using phased ipilimumab in conjunction with carboplatin and paclitaxel in patients with advanced or metastatic NSCLC concluded that ipilimumab should be given at a dose at 10 mg/kg, without significantly increased toxicity compared to a lower dose 47. Though data was limited, the authors concluded that the addition of ipilimumab showed encouraging antitumor activity. Data from phase III trials are pending for ipilimumab.

CheckMate‐063 was a phase II clinical trial testing nivolumab, a humanized IgG_4_ anti‐PD‐1 monoclonal antibody, in patients with stage IIIB/IV squamous NSCLC who had received at least two previous treatments, one of which was a platinum‐based doublet 48. A total of 15% of patients achieved a partial response and 30% maintained disease stability. A 6‐month progression‐free survival was 25.9% and 20.8% at 1 year. The favorable results were further demonstrated in CheckMate‐017, a phase III randomized trial comparing nivolumab to docetaxel in patients with stage IIIB/IV squamous NSCLC who had disease recurrence after being treated with a platinum‐containing regimen 49. Submission of a pretreatment tumor tissue sample was an enrollment criteria; membranous PD‐L1 expression was conducted retrospectively using the Dako 28‐8 rabbit anti‐PD‐L1 antibody. Positivity was defined as >1% of expression. The median overall survival was 9.2 months with nivolumab and 6.0 months with docetaxel. Objective response (defined as complete + partial response) was 20% in the nivolumab group compared to 9% in the docetaxel group. At the time of analysis, the median duration of response had not yet been reached in the nivolumab group (20.5+ months) compared to 8.4 months in the docetaxel group. The hazard ratios for progression‐free survival and overall survival in current/former smokers favored nivolumab over docetaxel. PD‐L1 expression was neither predictive of response to nivolumab nor prognostic of the trial efficacy endpoints. Nivolumab was then compared to docetaxel in a phase III trial in advanced nonsquamous non‐small cell lung cancer in CheckMate‐057. Again, nivolumab was shown to be superior to docetaxel with longer overall survival (median overall survival of 12.2 months compared to 9.4 months). Subgroups in which this benefit was not significant were patients who were never smokers as well as patients with EGFR (epidermal growth factor receptor)‐positive tumors 50.

Pembrolizumab, a humanized IgG_4_ antibody against PD‐1, was studied in the KEYNOTE‐001 phase I study at various doses and administration schedules to examine safety in a cohort of 495 patients that were either treated or untreated with locally advanced or metastatic NSCLC (irrespective of histology) 51. The investigators assessed membranous PD‐L1 expression of the tumor cells using the Dako 22C3 antibody. The best overall response was stable disease in 21.8% of patients. Overall response rate was 19.4% with the median duration of response of 12.5 months. A PD‐L1 expression of >50% was determined to be the cutoff based on ROC analysis. The overall response rate was 45.2% in patients with >50% PD‐L1 expression compared to 16.5% with 1–49% expression and 10.7% with <1% expression. PD‐L1 staining did not differ according to mutational status of EGFR, but was shown to be increased in patients with KRAS mutations. Pembrolizumab was then compared to docetaxel in KEYNOTE‐010, a randomized phase II/III trial 53. One of the key inclusion criteria of this study was that all patients must have PD‐L1‐expressing tumors (defined as >1% tumor cells as determined by the Dako 22C3 IHC assay). Patients were then randomized 1:1:1 to pembrolizumab 2 mg/kg, pembrolizumab 10 mg/kg, or docetaxel 75 mg/m^2^ every 3 weeks. Median overall survival for the pembrolizumab groups was 10.4 months and 12.7 months, 2 mg/kg and 10 mg/kg, respectively, and 8.5 months for docetaxel. The benefit was statistically significant for adenocarcinoma histology, but not for squamous histology. Overall response rate was 18% in both pembrolizumab groups compared to 9% in the docetaxel, with longer response seen in pembrolizumab (median response not yet reached) versus docetaxel (8 months).

Several findings from these landmark trials are worth discussing in further depth. Though PD‐L1 expression was assessed using the same assay and with the same cutoffs in CheckMate‐017 and Checkmate‐057, there was conflicting data regarding its role as a predictive and prognostic marker. While PD‐L1 expression was associated with response in patients with nonsquamous NSCLC, it carried no significance in patients with squamous NSCLC. It is unclear if this difference is due to modifiable factors (i.e., subjective interpretation of PD‐L1 positivity due to variability in IHC, subtle differences in performance of the assay) or true differences in the immune microenvironment between squamous and nonsquamous histologies. Patients with both squamous and nonsquamous NSCLC were enrolled into KEYNOTE‐001 and KEYNOTE‐010 and the predictive impact of PD‐L1 between the different histologies was not specifically assessed. In general, pembrolizumab was noted to be more efficacious in tumors with PD‐L1 expression >50%. However, there were again differences noted in terms of response to therapy between the histologies. In KEYNOTE‐010, the benefit of pembrolizumab over docetaxel in progression‐free survival was only statistically significant for adenocarcinoma but not for squamous cell carcinoma. This further supports the hypothesis that different interactions may be occurring in the squamous tumor microenvironment compared to the adenocarcinoma tumor microenvironment which can have implications on response to checkpoint inhibition and warrant further investigation. Finally, patients with mutant EGFR (present in adenocarcinomas) did not have a significantly improved response to nivolumab therapy or pembrolizumab as compared to docetaxel. It is unclear why this difference of response exists—one can hypothesize that tumors with EGFR mutations, which are not typically associated with significant smoking history, may have less mutational heterogeneity and therefore not respond as well to immunotherapy.

Based on these studies, the FDA granted approval to nivolumab in 2015 for the treatment of metastatic squamous and nonsquamous NSCLC. In the same year, the FDA also granted approval to pembrolizumab for the treatment of PD‐L1+ NSCLC. Studies using anti‐PD‐L1 antibodies in patients with metastatic and previously untreated NSCLC are ongoing with preliminary results showing favorable response and acceptable toxicity profile 54, 55 (Table 1).

**Table 1:** Trials with PD‐1 and PD‐L1 inhibitors as monotherapy in NSCLC

Agent	Trial (Phase)	Number of patients (*n*)	ORR (%)	Median PFS (months)	Median OS (months)
PD‐1 inhibitor					
Nivolumab	CheckMate017 49 (3)	272	20	3.5	9.2
Nivolumab	CheckMate057 50 (3)	582	19.0	2.3	12.2
Pembrolizumab	KEYNOTE‐001 51 (1)	495	19.4	3.7	12.0
Pembrolizumab	KEYNOTE‐010 53 (2/3)	1034	18^(2 mg/kg)^18.5^(10 mg/kg)^	3.9^(2 mg/kg)^4.0^(10 mg/kg)^	10.4^(2 mg/kg)^12.7^(10 mg/kg)^
PD‐L1 inhibitor					
Atezolizumab	NCT01475842 77 (1)^a^	88	21	ND	ND
MEDI4736	NCT01693562 54 (1/2)^a^	198	21/10^b^	ND	ND
Avelumab	NCT01772004 55] (1)^a^	184	12	2.9	ND

ORR, objective response rate; PFS, progression‐free survival; OS, overall survival; ND, not determined. NSCLC, non‐small cell lung cancer

a Abstract only.

b 21% in squamous histology, 10% in nonsquamous histology.

### Determinants of response to anti‐PD‐1/PD‐L1 therapy

There is growing evidence that anti‐PD‐1 and anti‐PD‐L1 therapy can lead to durable disease response in a subgroup of patients with NSCLC, even in those who were heavily pretreated for whom scant treatment options exist. However, it has not yet been elucidated what factors predict a favorable clinical outcome as only about 20% of patients treated with anti‐PD‐1/anti‐PD‐L1 monoclonal antibodies have meaningful response to therapy 44. We will summarize emerging data for host and tumor characteristics that may carry predictive value.

#### Antigenic burden

Genomic instability is the *sine qua non* of cancer and plays a critical role in cancer initiation and progression. These mutations have the potential to generate tumor‐specific antigens. Rizvi et al. have recently demonstrated through whole‐exome sequencing in NSCLC patients treated with pembrolizumab, that higher nonsynonymous mutation burden in tumors was associated with improved objective response, durable clinical benefit, and progression‐free survival 56. This observation is consistent with the hypothesis that the efficacy of anti‐PD‐1 therapy is largely related to recognition of neoantigens; these neoantigens result from various somatic mutations induced by carcinogens such as cigarette smoke. In their study, efficacy of therapy positively correlated with the molecular smoking signature (tobacco‐related mutagenesis), higher neoantigen burden (correlated with higher mutational burden), and mutations in certain DNA repair pathways. In KEYNOTE‐001, the response rate to pembrolizumab was 22.5% for current/former smokers compared to 10.3% in never smokers 51, further supporting that the higher mutational burden associated with smoking contributed to improved response to PD‐1 inhibition.

Le et al. found higher PFS and OS in patients with cancers harboring DNA mismatch‐repair deficiency when treated with pembrolizumab compared to those with tumors that were mismatch‐repair proficient 57. Mismatch‐repair deficient tumors expressed a mean of 1782 somatic mutations per tumor compared to 73 mutations per tumor in mismatch‐repair proficient tumors. Mutational burden in DNA repair pathways may be of special significance as a determinant of response to anti‐PD‐1 therapy because tumors harboring these mutations possess the capability to generate a high neoantigen burden. However, it is not entirely clear that generation of these neoantigens is a prerequisite for activity. Van Allen et al. recently showed, albeit in melanoma, that clinical benefit and overall survival in melanoma was likely related to a combination of factors that included higher mutational burden, but not necessarily related to neoantigen generation alone 58.

In addition to neoantigens generated by somatic mutations, there are also other aberrantly expressed antigens. Cancer/testis (CT) antigens are expressed in immune privileged tissues such as the testis and placenta, but aberrantly expressed in various cancers 59. CT antigens are highly immunogenic and are expressed in 10–30% of NSCLC 60. A limitation has been low affinity of these antigen‐reactive T cells and work is ongoing to optimize TCR affinity to these CT antigens 61.

#### Inflamed tumor microenvironment

An inflamed tumor phenotype seems to predict response to inhibition of the PD‐1/PD‐L1 pathway. Herbst et al. studied the characteristics of tumors in patients with advanced incurable cancers that did and did not respond to therapy with atezolizumab, a humanized IgG_1_ antibody against PD‐L1. Regressing lesions in responders displayed a dense immune infiltrate with elevated tumor IFN*γ* expression 62. RNA analysis from regressing lesions also showed a pattern indicative of activation of CD8+ T‐cell response. In contrast, tumors from nonresponding patients showed little or no tumor‐infiltrating immune cell infiltration, none to minimal tumoral expression of PD‐L1, or an immune infiltrate that resided solely in the outer edge of the tumor cell mass. Tumeh et al. analyzed samples from patients with metastatic melanoma before and after treatment with pembrolizumab. They found that higher CD8+ T‐cell densities at the invasive tumor margin was a strong predictor of response to pembrolizumab therapy 63. Furthermore, the response group showed higher expression of phosphorylated signal transducer and activator of transcription 1 (pSTAT1), a downstream effector of IFN*γ* binding, again validating the importance of the inflamed phenotype and response.

Zeng et al. conducted a meta‐analysis to assess the utility of TILs as both a prognostic factor and predictor of response to therapy in NSCLC patients 64. High levels of CD3+ T lymphocytes, CD4+ T lymphocytes, CD8+ T lymphocytes, and low levels of FOXP3+ Treg lymphocytes were all associated with a good prognosis for overall survival. Furthermore, Zeng et al. also found that a high FOXP3+/CD4+ ratio was a risk factor for disease recurrence, whereas a high CD4+/CD8+ ratio portended favorable overall survival. However, there is currently no standardized method to measure TIL levels or delineate TIL location. The studies included in this meta‐analysis were also not specific to immunotherapy. Therefore, while an inflamed tumor microenvironment, as determined by levels of TILs, suggests favorable response and survival, further studies are warranted to elucidate their role as a predictive biomarker.

#### Expansion of the TCR repertoire

Early studies using PCR to assess TCR repertoire in NSCLC indicated that antigen‐driven selection of T‐cell proliferation does indeed play a role in the immune response against NSCLC 65. Derniame et al. found a higher degree clonality of the TCR repertoire in the TILs compared to T cells found in adjacent healthy lung tissue in NSCLC patients 66. Specific TCR*β* clones in the tumor tissues were also present in the draining lymph nodes but not in the adjacent healthy lung tissue. Clonal expansion of certain TIL populations suggests tumor antigen‐specific T‐cell response. The study by Tumeh et al. also showed that a more restricted TCR*β* chain usage, reflecting a more clonal T‐cell population, correlated significantly with response to pembrolizumab 63.

#### Tumoral PD‐L1 expression

Carbognin et al. conducted a meta‐analysis of all trials in advanced melanoma, NSCLC, and genitourinary cancers in which patients received nivolumab, pembrolizumab, or atezolizumab and in which tumoral PD‐L1 expression was assessed by immunohistochemistry. The pooled data from the seven trials dedicated to NSCLC showed a statistically significant better overall response rate (ORR) in patients with PD‐L1‐positive tumors compared to those with PD‐L1‐negative tumors 67. Similarly, in an initial phase I study of nivolumab in advanced solid tumors including NSCLC, Topalian et al. also showed that tumoral PD‐L1 positivity defined as >5% expression predicted response to therapy 68. In their study, no patients with PD‐L1‐negative tumors (17 of 42) had an objective response to nivolumab. In the KEYNOTE‐001 study, tumor positivity for PD‐L1 as defined as >50% expression correlated with likelihood of response to pembrolizumab 52. However, not all studies have consistently shown that elevated tumoral PD‐L1 expression predicted response to PD‐1 inhibitor therapy 50, 69 and therefore its prognostic value is unclear.

#### PD‐1 expression by TILs

Thommen et al. showed in an in vitro study of NSCLC that the efficacy of nivolumab in restoring T‐cell effector function (as measured by expression of cytokines IL‐2, IFN*γ*, and TNF*α*) was inversely correlated with the percentage of high PD‐1‐expressing T cells 70. These PD‐1^hi^ T cells had significantly higher expression of co‐inhibitory receptors such as CTLA‐4 and TIM‐3. The authors suggested that high expression of PD‐1 may represent an exhausted T‐cell phenotype that cannot be recovered by PD‐1 blockade alone. Further staining on specimens from the study by Topalian et al. suggested that there was no correlation between TIL expression of PD‐1 and response to PD‐1 inhibitors 71.

### Future directions

An active area of research is improving tumor immunogenicity, thereby increasing the likelihood of response to therapy with immune checkpoint inhibitors. Radiation therapy (RT) is commonly employed in the treatment of NSCLC and there is increasing evidence that it can modulate the immune response and assist in overcoming the immune‐suppressing hurdles effected by tumor cells. RT has been shown to enhance APC activity by inducing the translocation of calreticulin (CRT) to the plasma membrane of the dying tumor cell. CRT couples with MHCI to enhance antigen presentation and plays an important role in the phagocytosis of tumor cells by APCs 72. In lung cancer, radiation therapy can also induce release of proinflammatory cytokines such as TNF*α* and enhance T‐cell recruitment through increasing expression of adhesion molecules and homing chemokines 73. Local high‐dose irradiation in a mouse tumor model also significantly increased PD‐L1 expression by the tumor cells 74.

The CTLA‐4 (including other B7 family proteins) and PD‐1/PD‐L1 pathways are just a few among many others that play a key role in T‐cell immunomodulation. Active research is underway to either enhance the effects of existing stimulatory pathways (OX40/OX40L, CD40/CD40L) or disinhibit inhibitory pathways (VISTA, Tim‐3, LAG‐3). The OX40/OX40L pathway promotes effector T‐cell expansion and proliferation and has been shown to downregulate Treg cells in mouse models. A phase I study of an agonist monoclonal antibody against OX40 in patients with advanced cancers showed stable disease or tumor regression in over half of the patients, though none achieved a partial response by RECIST criteria 75. LAG‐3 is closely related to CD4 and downregulates T‐cell activation through modulation of TCR signaling; it has also been shown to upregulate Treg function. A phase I clinical trial using a LAG‐3 inhibitor in patients with advanced renal cell carcinoma showed improved progression‐free survival in patients receiving higher doses and that the drug (IMP321) was well tolerated 76. Another approach that has not been well explored in NSCLC is the use of combination immunotherapy, as currently is standard in the treatment of metastatic melanoma with both ipilimumab and nivolumab. One can conceive that in NSCLC, there may be additive benefits of using drugs that augment T‐cell proliferation and downregulate T‐cell inhibition.

NSCLC is a molecularly heterogeneous disease, and presents a large mutational load that likely encodes a large number of potential neoantigens. Yet, this cancer evades recognition by the immune system. Novel therapies that target immune checkpoints have for the first time demonstrated responses in these cancers, with some patients exhibiting durable responses weeks to months after discontinuing therapy. Importantly, these are the first therapeutic agents to offer significant benefit in smoking‐related cancers that are extremely heterogenous. However, it is crucial to identify factors associated with response and those involved in maintaining response durability. There is still much that is unknown regarding biomarkers that can predict response to immunotherapy. Furthermore, the optimal method(s) to monitor response are unclear. There are no routine blood tests akin to tumor markers in certain malignancies that can be used as a surrogate of disease response. Imaging is not necessarily reliable as there are variable patterns of response 77. Finally, identifying the specific T‐cell subsets involved in these antitumor responses will pave the way for adoptive T‐cell‐based therapies that can be individualized for patients, ultimately improving the availability of therapies for patients with NSCLC who continue to have limited options in this era of molecular‐targeted therapy.

## Conflict of Interest

The authors do not have any conflicts of interest to disclose.
